# Chemotherapy-induced apoptosis, autophagy and cell cycle arrest are key drivers of synergy in chemo-immunotherapy of epithelial ovarian cancer

**DOI:** 10.1007/s00262-018-2199-8

**Published:** 2018-08-24

**Authors:** John Wahba, Marina Natoli, Lynsey M. Whilding, Ana C. Parente-Pereira, Youngrock Jung, Stefania Zona, Eric W.-F. Lam, J. Richard Smith, John Maher, Sadaf Ghaem-Maghami

**Affiliations:** 1 Department of Surgery and Cancer, Institute of Reproductive and Developmental Biology, Imperial College London, Hammersmith Hospital Campus, Du Cane Road, London, W12 0NN UK; 2 King’s College London, Guy’s Hospital Campus, London, UK

**Keywords:** CAR T cells, Ovarian cancer, Apoptosis, In vivo models, Combination chemo-immunotherapy, Anti-PD-1

## Abstract

Epithelial ovarian cancer (EOC) is the most lethal of all gynecological malignancies in the UK. Recent evidence has shown that there is potential for immunotherapies to be successful in treating this cancer. We have previously shown the effective application of combinations of traditional chemotherapy and CAR (chimeric antigen receptor) T cell immunotherapy in in vitro and in vivo models of EOC. Platinum-based chemotherapy synergizes with ErbB-targeted CAR T cells (named T4), significantly reducing tumor burden in mice. Here, we show that paclitaxel synergizes with T4 as well, and look into the mechanisms behind the effectiveness of chemo-immunotherapy in our system. Impairment of caspase activity using pan-caspase inhibitor Z-VAD reveals this chemotherapy-induced apoptotic pathway as an essential factor in driving synergy. Mannose-6-phosphate receptor-mediated autophagy and the arrest of cell cycle in G2/M are also shown to be induced by chemotherapy and significantly contributing to the synergy. Increased expression of PD-1 on T4 CAR T cells occurred when these were in culture with ovarian tumor cells; on the other hand, EOC cell lines showed increased PD-L1 expression following chemotherapy treatment. These findings provided a rationale to look into testing PD-1 blockade in combination with paclitaxel and T4 immunotherapy. Combination of these three agents in mice resulted in significant reduction of tumor burden, compared to each treatment alone. In conclusion, the mechanism driving synergy in chemo-immunotherapy of EOC is multifactorial. A deeper understanding of such process is needed to better design combination therapies and carefully stratify patients.

## Introduction

Epithelial ovarian cancer (EOC) is the fifth commonest malignancy in women with up to a quarter of a million new diagnoses per year worldwide [1, 2]. It presents at a late stage in up 75% of the cases [3]. Overall, 5-year survival has improved over the past few years but it is still low at around 40% and even less for advanced disease [4]. Current management of the disease is largely age- and stage-dependent and involves debulking surgery followed by (or sometimes preceded by) platinum-based chemotherapy, with or without a taxane. Poor survival is thought to be attributed to various factors including the non-specific symptoms of the disease, the advanced disease stage at diagnosis and acquisition of chemo-resistance following treatment [5–8]. There is therefore a need for novel, more targeted and personalized therapies.

Immunotherapy is an exciting new avenue and its use is gaining huge momentum in oncology with adoptive T cell therapy showing great promise in some cancers such as metastatic melanoma [9].

We have previously shown the successful use of chimeric antigen receptor (CAR) T cells in EOC as well as in other tumor models, in vitro and in vivo [10, 11]. We developed a CAR (T4) targeting ErbB homo- and hetero-dimers [12], which have been shown to be upregulated in EOC [13–16]. T4 cells were able to mediate effective anti-tumor activity in EOC in vitro and in our ovarian cancer mouse model [10]. Combining T4 cells with carboplatin or cisplatin resulted in even better killing of tumor cells and this interaction is thought to be synergistic [10]; however, the mechanism for this interaction remains unclear.

There is growing evidence that chemotherapy may be immunomodulatory, depending on the dosage and schedule administered [17–19]. It can alter the tumor microenvironment by modulating tumor antigen expression, antigen processing and T cell activation [20, 21].

Combination of different therapeutic strategies is becoming the way forward in the field of cancer immunotherapy. A relevant example is that of immune checkpoint inhibitors [22, 23].

A better understanding of the mechanisms that might drive synergy and determine clinical success is urgent and necessary to better carefully design such combination strategies and predict patient response.

Having previously shown a synergistic relationship between platinum-based agents ad T4 cells, we investigated if paclitaxel, the other main chemotherapeutic agent used in the treatment of EOC, would show a similar effect. We hypothesized that the effects of chemotherapy on processes such as apoptosis, autophagy and cell cycle are responsible for sensitization of EOC to immunotherapies such as T4 CAR T cells. We investigated the effect of impairing such processes in an effort to elucidate the mechanisms for the observed synergistic chemo-immunotherapy interactions. Additionally, our findings provided a rationale to look into augmenting this chemo-immunotherapy combination with PD-1 blockade and we tested this novel three-agent approach in our in vitro and in vivo EOC models.

## Materials and methods

### Cell culture

Human epithelial ovarian cancer cell lines were grown in RPMI-1640 media (Sigma-Aldrich, Poole, UK) with 10% FBS (Sigma-Aldrich, Poole, UK) and l-glutamine 200 mM, penicillin 10,000 units, streptomycin 10 mg/mL solution (Sigma-Aldrich, Poole, UK). The firefly luciferase (luc)-expressing SKOV-3 cell line (SKOV-3-luc) was purchased for this work from Cell Biolabs Inc. (California, USA). OVCAR-4 cell line was available in the laboratory of Sadaf Ghaem-Maghami. The retroviral vector packaging cell line, PG13, which either expressed the sequence for T4 or mock constructs, was maintained in DMEM supplemented with 10% FBS, l-glutamine 200 mM, penicillin 10,000 units, streptomycin 10 mg/mL solution and ciprofloxacin 5 µg/mL (CIPRO; Bayer, New Jersey, USA). This packaging cell line was kindly supplied by the laboratory of John Maher (King’s College London, UK) [10]. The J591 scFv (used to generate the mock CAR) was supplied under MTA to John Maher by Neil Bander (Cornell University, New York). All cells were cultured in 37 °C with 5% CO_2_.

### Retroviral transduction of human T cells

Transduction of human T cells, isolated from healthy volunteers’ blood, was performed as previously described by Davies et al. [12].

### Flow cytometry

Antibodies used for the analysis of cell surface markers were PE-conjugated mouse anti-human Mannose-6-phosphate receptor (M6PR) antibody (Abcam, Cambridge, UK) for tumor cells, PE-conjugated mouse anti-human CD279 (PD-1, BD Biosciences Pharmingen, California, USA) and APC-conjugated mouse anti-human CD3 (BD Biosciences Pharmingen, California, USA) for T cells.

For detecting apoptosis, tumor cells were stained with FITC-Annexin V (BD Biosciences Pharmingen, California, USA) and 7-AAD (7-amino-actinomycin; BD Biosciences Pharmingen, California, USA).

For cell cycle analysis, tumor cells, following the addition of the desired drug treatments, were collected by trypsinization and then centrifuged. The cell pellet was resuspended in 300 µL ice-cold 70% ethanol and incubated in the dark at 4 °C for 24 h. The cell pellet was then resuspended in propidium iodide 1 mg/mL (Sigma-Aldrich, Poole, UK), RNase A 1 mg/mL (Invitrogen, Paisley, UK) and PBS, and incubated for 30 min.

10,000 events were acquired on a FACSCalibur flow cytometer (Becton Dickinson Immunocytometry Systems, California, USA) with CellQuest Pro Software version 4.0.2 (Becton Dickinson, California, USA). FlowJo software version 6.4.7 (Tree Star Inc, Oregon, USA) was used for analysis.

For cell cycle analysis, gating was performed using forward and side scatter dot plots on the tumor cell population. A second gate was setup to exclude doublets on a dot-plot of pulse width versus pulse area. PI fluorescence was plotted against cell count to distinguish between the various phases of the cell cycle.

For the analysis of tumor cell surface markers after tumor:T cell co-culture, gating was performed using forward and side scatter dot plots on the tumor cell population, avoiding the T cell population, and CD3-positive cells were excluded.

### Cell viability assay

Cell viability was quantified using a MTT (Sigma-Aldrich, Poole, UK) assay. Tumor cells were seeded onto a 96-well plate at a density of 1 × 10^4^ tumor cells per well and cultured for 24 h. Paclitaxel (Teva, Castleford, UK) or carboplatin (Fresenius Kabi Oncology, Hampshire, UK) were added the following day, diluted in RPMI media at the required concentrations. Other drugs such as 25 ∝M Z-VAD (carbobenzoxy-valyl-alanyl-aspartyl-[*O*-methyl]-fluoromethylketone: R&D), 5 mM 3-methyladenine (3-MA; Sigma-Aldrich, Poole, UK) or Thiostrepton (Sigma-Aldrich, Poole, UK) were added at this stage. Cells were incubated for a further 48 h, after which the supernatant was removed and T cells were added to the wells. For experiments looking at the effect of PD-1 antibody, T cells were incubated with anti-PD-1 antibody 20 µg/µL (BMS-936558; Bristol-Myers-Squibb, New York, USA) for 1 h before being added to the tumor cells and being co-cultured for 24 h. The supernatant was removed and MTT 500 µg/mL (Sigma-Aldrich, Poole, UK) was added for 2–3 h. Formazan crystals were resuspended in 90 µL of dimethyl sulfoxide (DMSO; Sigma-Aldrich, Poole, UK). Absorbance was measured at a wavelength of 570 nm using an OPTImax tunable microplate reader programmed with Softmax Pro software version 4.2 (Molecular Devices Corporation, California, USA). Tumor cell viability was calculated as follows: (absorbance of monolayer cultured with T cells/absorbance of untreated monolayer alone) × 100.

### Caspase activity

Apo-Tox-Glo Triplex kit (Promega, California, USA) was used to measure tumor cell viability and caspase-3/7 activity following the manufacturer’s protocol. Both fluorescence (for cell viability) and luminescence (for caspase activation) were measured on a LUMIstar OPTIMA microplate reader programmed with OPTIMA software version 2.20R2 (BMG Labtech, Aylesbury, UK) and caspase activity was normalized to cell viability.

### Quantitative polymerase chain reaction (q-PCR)

RNA extraction and cDNA conversion were performed using RNeasy Micro Kit (Qiagen, California, USA) and High-Capacity cDNA Reverse Transcription Kit (Applied Biosystems, Paisley, UK), respectively, as per the manufacturers’ instructions. Real-time PCR was performed using 7900HT Real-Time PCR System (Applied Biosystems, Paisley, UK) and analyzed using the 2 (-delta delta C(T)) method. qPCR primers sequences were as follow: GAPDH sense 5′-AGCCACATCGCTCAGACAC-3′, antisense 5′-GCCCAATACGACCAAATCC-3′; M6PR sense 5′-CCACACTTCCACAGATGTTGA-3′, antisense 5′-CCGAGCTGTGCAGTTATACAT-3′.

### Enzyme-linked immunosorbent assay (ELISA)

Conditioned cell culture medium was analyzed for human IFN-γ and Granzyme B using the Human IFN gamma ELISA Ready-SET-Go (eBiosciences, California, USA) and the Human Granzyme B Platinum ELISA (eBiosciences, California, USA) kits, respectively, according to manufacturer’s protocol.

### Confocal imaging

SKOV-3-luc cells were seeded on 13 mm diameter glass cover slips at a seeding density of 3 × 10^4^ cells per slide and were treated with paclitaxel or carboplatin with and without 3-MA for 48 h as described previously. The cells were fixed with 4% paraformaldehyde for 5 min, washed with PBS containing 1% FBS and stained with mouse anti-human M6PR primary antibody (Abcam, Cambridge, UK) and Alexa Fluor 488 Goat anti-mouse secondary antibody (Life Technologies, Paisley, UK). The cells were mounted on a glass slide using ProLong Gold Antifade Mountant (Life Technologies, Paisley, UK) and imaged using a Leica TCS SP5 Confocal Laser Scanning Microscope.

### In vivo studies

SCID Beige mice were inoculated with 1 × 10^6^ SKOV-3-luc tumor cells intraperitoneally (i.p.) and tumor engraftment was confirmed 4 days later using bioluminescence imaging (BLI; IVIS Lumina Series III, PerkinElmer, Massachusetts, USA). The tumors were allowed to graft for 21 days before treatment was initiated. There were five mice in each group (except for the PBS group where there were only two mice). One group was treated with paclitaxel (10 mg/kg) on day 21, followed by 2.5 × 10^6^ T4 cells on day 23 and anti-PD-1 antibody (10 mg/kg) every 48 h. The other groups received either PBS, paclitaxel alone on day 21 followed by PBS, T4 cells alone on day 23, paclitaxel on day 21 followed by T4 cells on day 23, or T4 cells on day 23 followed by anti-PD-1 antibody every 48 h. BLI was performed weekly to assess tumor response to treatment and Living Image software (PerkinElmer, Massachusetts, USA) was used for analysis. Mice were injected i.p. with 150 mg/kg d-Luciferin (Caliper life Sciences, Massachusetts, USA) and imaged whilst being anaesthetized with isoflurane (Isoflo; Abbott Laboratories Ltd, Kent, UK). Image acquisition was conducted on a 25-cm field of view with medium binning and auto-exposure. Animals were inspected daily for signs of ill health. Mice were culled when experimental endpoints had been achieved or if they became symptomatic as a result of tumor burden.

### Statistical analysis

Statistical analysis was carried out using Graphpad Prism software version 6.00 (Prism, California, USA) using a two-way ANOVA test to compare mean differences between two independent variables. Results are expressed as mean ± SEM based on three or more repeats. *p* < 0.05 was considered statistically significant.

## Results

### Paclitaxel, as well as carboplatin, enhances sensitivity to T4 immunotherapy

**Fig. 1:**
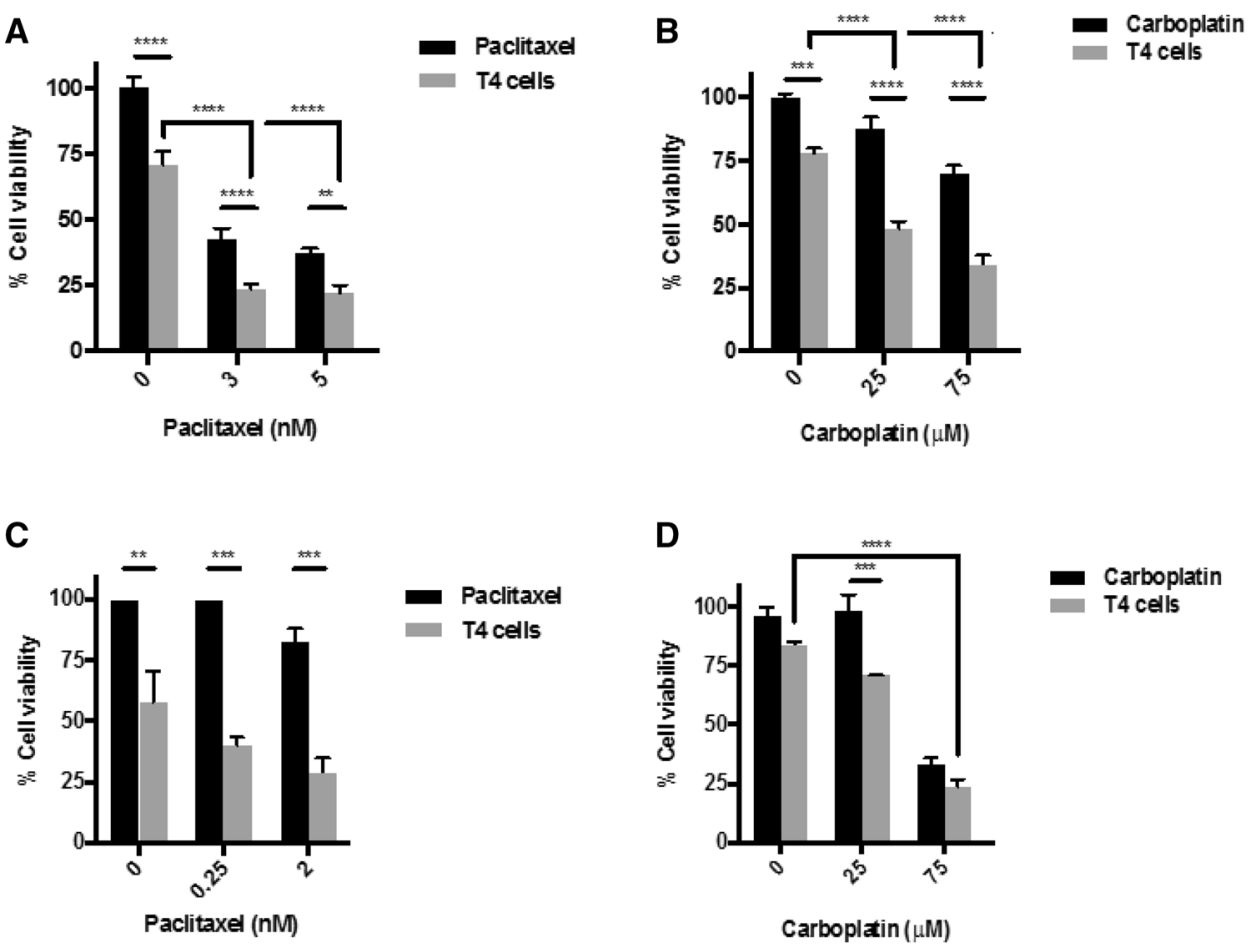
In vitro assessment of anti-tumor activity of T4 cells against ovarian tumor cell lines in combination with paclitaxel or carboplatin. **a, b** SKOV-3-luc cell viability following combination treatment of T4 and paclitaxel (**a**) or carboplatin (**b**). **c, d** OVCAR-4 cell viability following combination treatment of T4 and paclitaxel (**c**) or carboplatin (**d**). Data show mean ± SEM using T cells from separate donors (*n* = 6); **p < 0.01, ***p < 0.001, ****p < 0.0001

To determine the effect of combining chemotherapy, in the form of carboplatin or paclitaxel, with T4 immunotherapy, tumor cells were treated with either drug for a period of 48 h followed by the addition of T4 cells for a further 24 h. Cell viability was measured by MTT assay, as described above. Treatment of SKOV-3-luc cells with paclitaxel (Fig. 1a) or carboplatin (Fig. 1b) followed by the addition of T4 cells resulted in a significant reduction in tumor cell viability. At each dose tested, the results show a significant reduction in cell viability with combination treatment compared to individual therapies. This same effect was seen in OVCAR-4 (Fig. 1c, d). The doses used for both drugs were on either side of the IC-50 for the cell lines. Interestingly, the significant reduction in cell viability is even more prominent at the lower doses, compared to the higher doses. The effect was replicated in our in vivo tumor model where the combination of paclitaxel and T4 immunotherapy resulted in a significant reduction in tumor burden (Fig. 6b).

### Impairment of apoptotic caspase pathways partially reverses synergistic killing of ovarian cancer cells

**Fig. 2:**
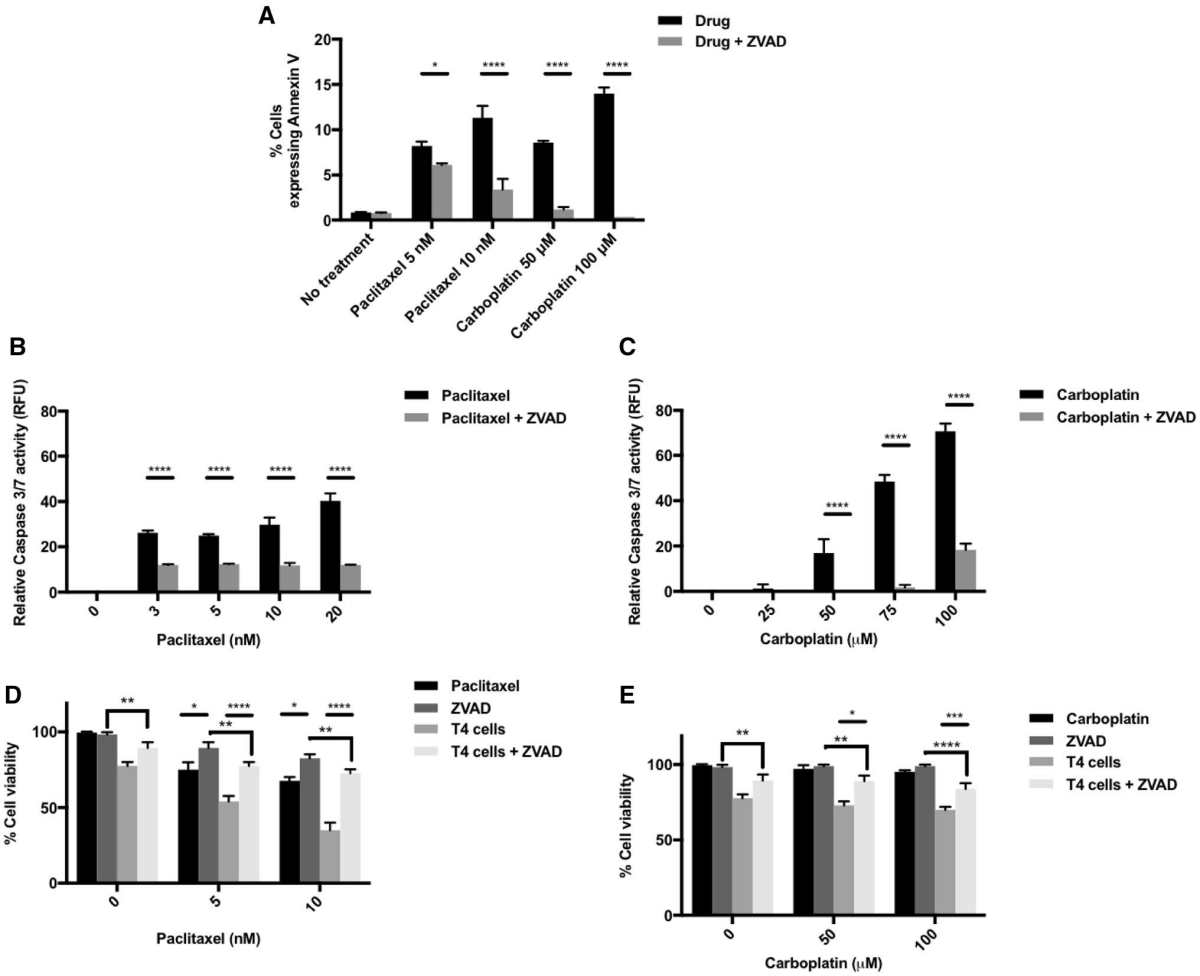
Induction of apoptosis by chemotherapy as a possible mechanism for synergy between chemotherapy and T4 cells. **a** Flow cytometry analysis of Annexin V expression on SKOV-3-luc tumor cell surface following treatment with various doses of paclitaxel or carboplatin ± the pan-caspase inhibitor Z-VAD. **b, c** Caspase 3/7 activity following treatment of SKOV-3-luc with various doses of paclitaxel (**b**) or carboplatin (**c**) ± Z-VAD. **d, e** SKOV-3-luc cell viability following combination treatment of T4 and paclitaxel (**d**) or carboplatin (**e**) ± Z-VAD. Data show mean ± SEM (*n* = 3); *p < 0.05, ***p < 0.001, ****p < 0.0001

To investigate the mechanisms by which chemotherapy appears to sensitize ovarian cancer cell lines to T4 immunotherapy, we assessed tumor cell surface expression of annexin V and caspase 3/7 activity as markers of apoptosis. Figure 2a shows a significant increase in the tumor cell surface expression of annexin V after treatment with chemotherapy as measured by flow cytometry; furthermore, there was a significant increase in tumor cell caspase 3/7 activity with increasing doses of chemotherapy (Fig. 2b, c). Such increase in cell surface annexin V and caspase activity was reversed by treatment with Z-VAD [24], a pan-caspase inhibitor which binds irreversibly to caspase proteases, inhibiting the induction of apoptosis (Fig. 2a–c).

To test our hypothesis that chemotherapy-induced apoptosis contributes to the synergistic effect of our combination therapy, SKOV-3-luc cells were treated with chemotherapy and Z-VAD for 48 h followed by treatment with T4 cells (Fig. 2d, e). Z-VAD caused a partial reversal in the reduction in tumor cell viability induced by combination treatment with chemotherapy and T4 cells. The reversal was almost complete; however, the fact that tumor cell viability did not entirely return to base levels might suggest that apoptosis is not the only mechanism of sensitization to T4 immunotherapy.

### Shuttling of M6PR (mannose-6-phosphate receptor) to the ovarian tumor cell surface is necessary for synergistic killing by chemo-immunotherapy

**Fig. 3:**
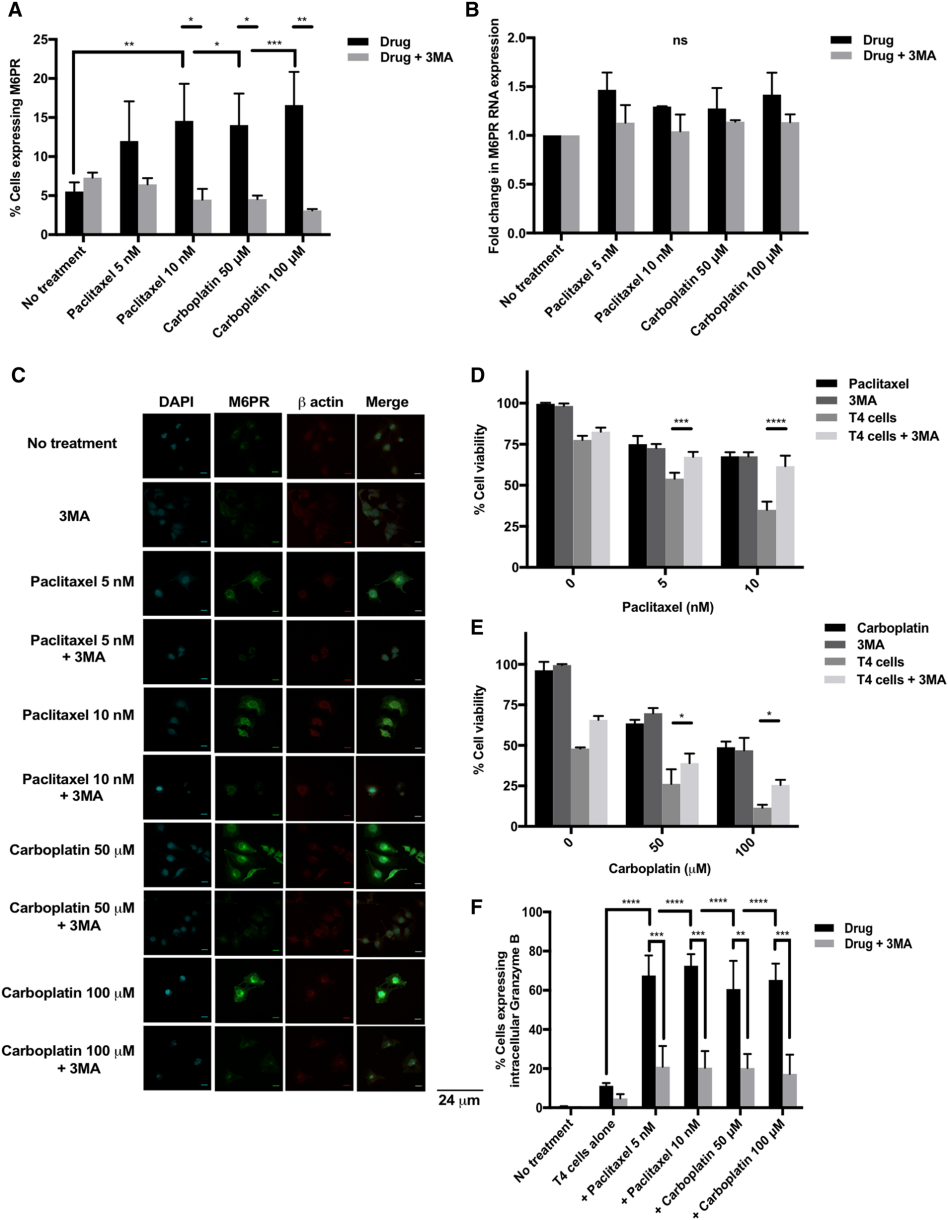
Role of tumor cell surface Mannose-6-Phosphate receptor (M6PR) in the synergy between chemotherapy and T4 cells. **a** Flow cytometry analysis of M6PR expression on SKOV-3-luc tumor cell surface following treatment with paclitaxel or carboplatin ± 3-Methyladenine (3MA). **b** M6PR mRNA quantification from SKOV-3-luc cells treated with chemotherapy ± 3MA. **c** Immunofluorescence imaging of SKOV-3-luc cells treated with chemotherapy ± 3MA showing cell surface M6PR, β actin cytoskeleton and nuclear staining (DAPI). **d, e** SKOV-3-luc cell viability following combination treatment of T4 and paclitaxel (**d**) or carboplatin (**e**) ± 3MA. **f** Flow cytometric analysis of intracellular expression of Granzyme B in SKOV-3-luc following treatment with paclitaxel or carboplatin ± 3MA. Data show mean ± SEM (*n* = 3 for A-B, E-G, *n* = 1 for C-D); *p < 0.05, **p < 0.01, ***p < 0.001, ****p < 0.0001, *ns* not significant

It has been suggested in the literature that chemotherapy induces an upregulation in tumor cell surface M6PR, facilitating cytotoxic killing by T cells. Figure 3a shows that there is indeed a significant upregulation in M6PR surface expression on SKOV-3-luc cells following treatment with chemotherapy. However, mRNA levels (Fig. 3b) remain largely unchanged which suggests the potential shuttling of M6PR protein from the cytoplasm to the cell surface rather than de novo synthesis. This hypothesis was confirmed by immunofluorescence; Fig. 3c shows upregulation of M6PR on the tumor cells’ surface following the addition of chemotherapy.

3-methyladenine (3-MA) is an autophagy inhibitor which blocks autophagosome formation through inhibition of type III PI3K [25, 26]; the process which leads to shuttling of M6PR to the cells’ surface [27]. As expected, the addition of 3-MA to chemotherapy resulted in a downregulation of tumor cell surface M6PR (Fig. 3a, c); mRNA levels did not change (Fig. 3b).

3-MA was further used in combination with chemotherapy and T4 cells to assess the contribution of the shuttling of M6PR in the mechanisms of chemo-sensitization to T4 immunotherapy (Fig. 3d, e). The addition of 3-MA to chemotherapy alone did not cause a change in SKOV-3-luc cell viability, as expected, when there were no T cells present. However, 3-MA caused a significant reversal in the reduction in tumor cell viability induced by combination treatment with chemotherapy and T4 cells, suggesting that exposure of M6PR to the tumor cell surface plays an essential role in synergistic killing.

Additionally, there was a significant increase in tumor intracellular Granzyme B expression as measured by flow cytometry following treatment with chemotherapy and T4 cells (Fig. 3f). This was significantly reversed with 3-MA, further supporting the role of M6PR in facilitating cytotoxic killing by T cells.

### Induction of G2/M arrest in ovarian cancer cell lines enhances sensitivity to T4 immunotherapy

**Fig. 4:**
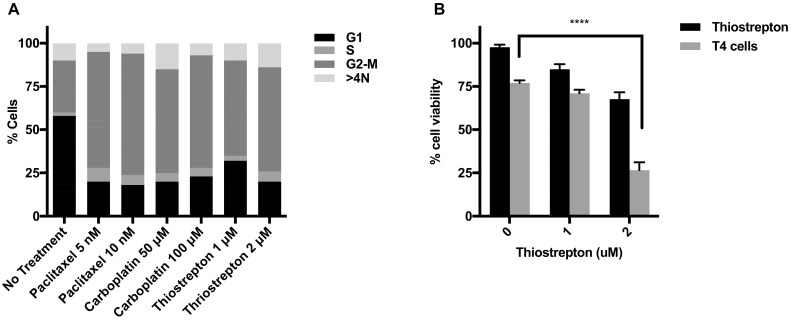
G2/M arrest enhances anti-tumor activity of T4 cells. **a** Flow cytometric cell cycle analysis of SKOV-3-luc treated with various doses of paclitaxel, carboplatin or Thiostrepton. **b** SKOV-3-luc cell viability following combination treatment of Thiostrepton ± T4. Data show mean ± SEM; ****p < 0.0001

Both paclitaxel and carboplatin are known to share a common mechanism that is the induction of G2/M arrest; which was observed in vitro in our ovarian cancer cells (Fig. 4a). Thiostrepton is a cyclic peptide antibiotic which inhibits protein synthesis by blocking the binding of GTP to the 50S ribosomal subunit [28] and specifically targeting the G2/M regulatory transcription factor FOXM1 [29]. Treatment with Thiostrepton also induced a G2/M arrest in ovarian tumor cells (Fig. 4a). To assess the contribution of G2/M cell cycle on the synergy seen between chemotherapy and T4 immunotherapy, SKOV-3-luc cells were treated with Thiostrepton for 48 h followed by T4 cells treatment. Figure 4b shows a significant reduction in tumor cell viability when cells were treated with Thiostrepton and T4 cells, an effect which is similar to combination of carboplatin/paclitaxel and T4 immunotherapy. This result supports a role for G2/M arrest in enhancing ovarian cancer cells sensitivity to immunotherapy.

### PD-1 blockade augments chemo-immunotherapy of ovarian cancer

**Fig. 5:**
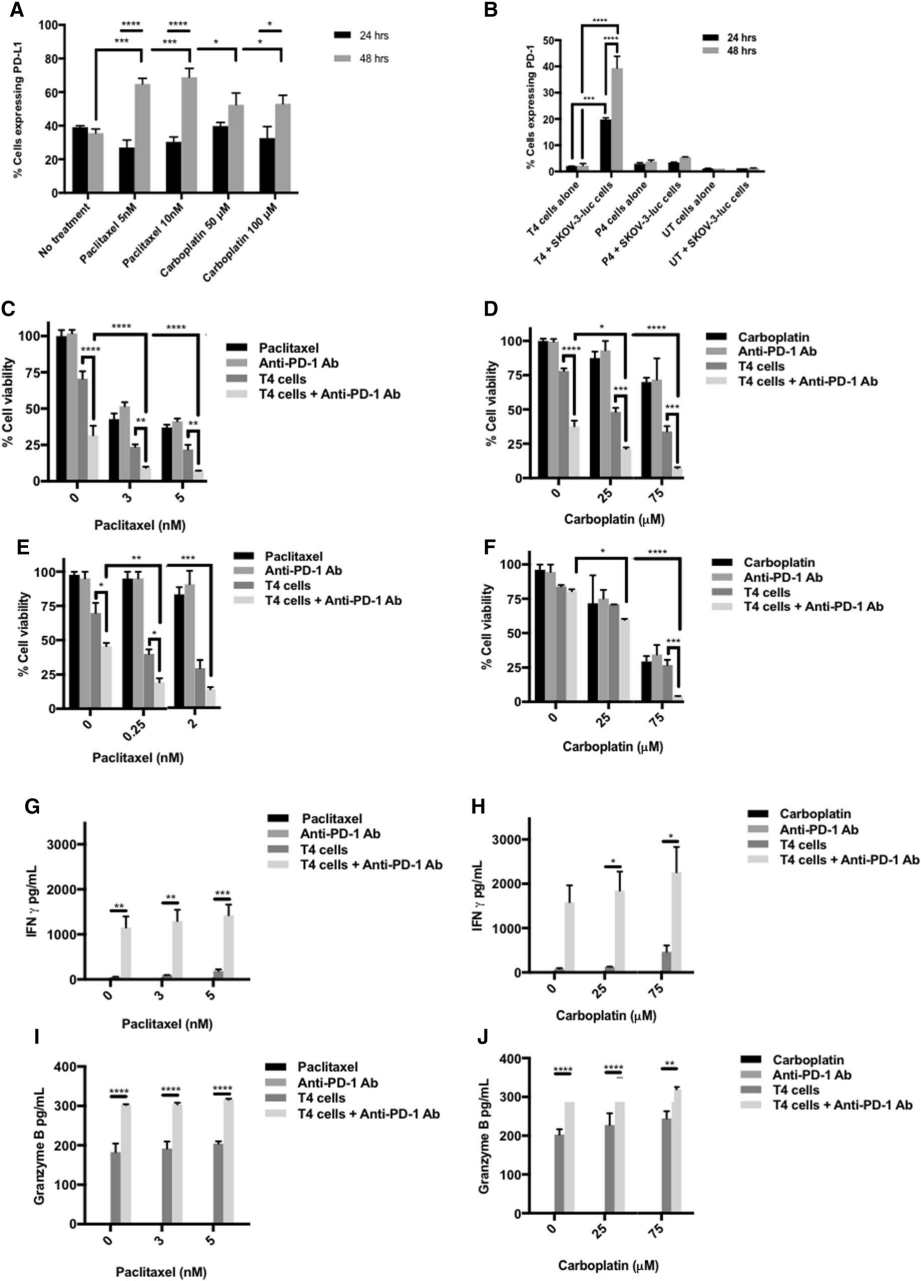
PD-1 blockade further enhances the synergy between chemotherapy and T4 cells. **a** Flow cytometric analysis of surface PD-L1 expression on SKOV-3-luc after exposure to chemotherapy for 24 and 48 h. **b** Flow cytometric analysis of surface PD-1 expression on T4 cells alone or after co-culture with SKOV-3-luc cells for 24 and 48 h (*P4* mock construct; *UT* untransduced T cells). **c, d** SKOV-3-luc cell viability following combination treatment of T4 and paclitaxel (**c**) or carboplatin (**d**) ± anti-PD-1 antibody. **e, f** OVCAR-4 cell viability following combination treatment of T4 and paclitaxel (**e**) or carboplatin (**f**) ± anti-PD-1 antibody. **g, h** IFN γ concentration in supernatants from SKOV-3-luc cells treated with paclitaxel (**g**) or carboplatin (**h**) ± T4 cells ± anti-PD-1 antibody. **i, j** Granzyme B concentration in supernatants from SKOV-3-luc cells treated with paclitaxel (**i**) or carboplatin (**j**) ± T4 cells ± anti-PD-1 antibody. Data show mean ± SEM using T cells from separate donors (*n* = 3); *p < 0.05, **p < 0.01, ***p < 0.001, ****p < 0.0001

Interestingly, 48 h treatment with either carboplatin or paclitaxel significantly increased cell surface expression of PD-L1 in SKOV-3 (Fig. 5a). Moreover, following the co-culture of SKOV-3-luc cells with T4 cells, there was a significant upregulation in the surface expression of PD-1 on T4 cells at 24 and 48 h (Fig. 5b). This did not occur with either P4 cells (mock construct) or untransduced cells (activated T cells which have not been genetically modified). Considering the immunosuppressive nature of the PD-1 pathway in cancer, we investigated whether using a fully human monoclonal PD-1 blocking antibody would aid chemo-immunotherapy interactions in our system.

**Fig. 6:**
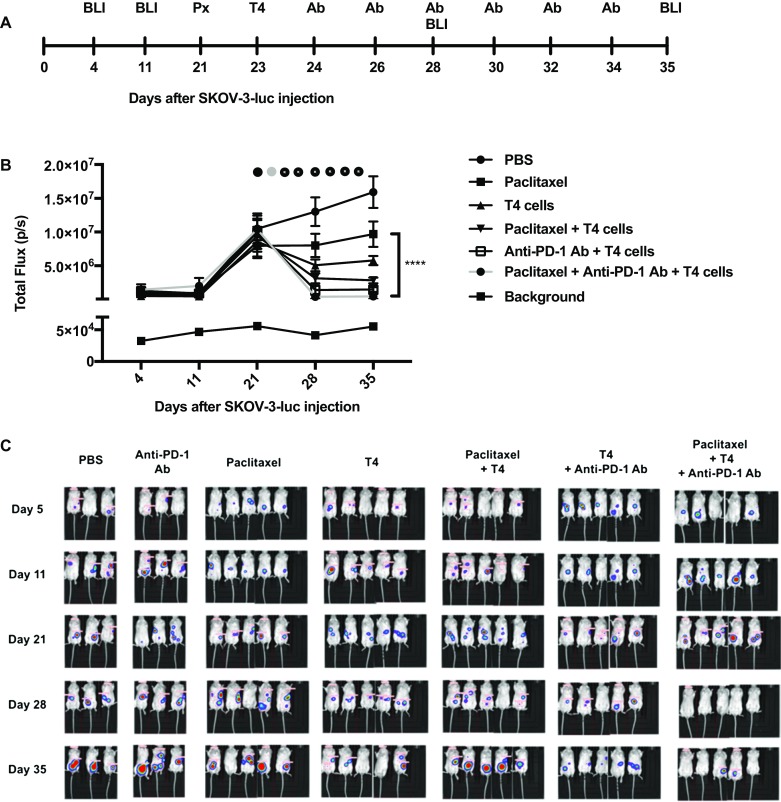
In vivo testing of using an anti-PD-1 antibody with T4 immunotherapy in mice-bearing SKOV-3-luc intraperitoneal tumor xenografts following treatment with paclitaxel. **a** Summary of the experimental protocol. **b** Serial BLI of mice before and after treatment (black circle—paclitaxel, gray circle—T4 cells, black circle with gray dot—anti-PD-1 antibody). Data show mean ± SEM of 5 mice in each group, with background light emission detected from a tumor-free mouse; ****p < 0.0001 (day 35). **c** BLI images of all the mice before and after treatment, shown on the same scale

Treatment with anti-PD-1 in combination with chemotherapy and T4 immunotherapy resulted in a significant reduction in SKOV-3-luc (Fig. 5c, d) and OVCAR-4 cell viability (Fig. 5e, f) compared with chemotherapy alone, T4 alone, and importantly, combination chemotherapy-T4 treatment. There was no change in cell viability when PD-1 blockade and chemotherapy were given with no T cells. Functionally, PD-1 blockade resulted in a significant increase in production of IFN-γ (Fig. 5g, h) and Granzyme B (Fig. 5i, j) by T4 cells, compared with T4 cells alone. In vivo, mice treated with paclitaxel, T4 cells and repeated doses of anti-PD-1 antibody had a significant reduction in their tumor burden compared to mice receiving paclitaxel alone, anti-PD-1 antibody alone, or T4 cells alone (Fig. 6b, c). The tumor burden in the mice that received triple treatment was lower at day 33 than in the mice that received paclitaxel and T4 cells. However, this difference was not significant.

## Discussion

In this study, we have shown that combination treatment of ovarian cancer cells with paclitaxel or carboplatin and adoptive T cell therapy in the form of ErbB-targeted CAR T cells resulted in an enhanced anti-tumor effect; greater than when either therapy was used alone. Our previous work showed a clear synergy between platinum-based agents and T4 immunotherapy in vitro and in our in vivo mouse model of ovarian cancer. The current work carries this forward and shows that paclitaxel, another first-line agent that is commonly used in the management of ovarian cancer, similarly synergizes with T4 immunotherapy. In this study, mice harboring intraperitoneal tumors were treated with a low dose of paclitaxel followed by administration of T4 cells. This resulted in a substantial regression in the tumors.

Though it is tricky to translate the chemotherapy doses used on cell lines in this work to human-equivalent ones, based on where these doses lie in relation to the IC50, it would be reasonable to assume that they are ‘low’ doses.

It is worth noting that there was not much tumor regression following the administration of paclitaxel alone or T4 cells as ‘low toxicity’ small dose and number were used, respectively, in view of a potential translation into the clinic.

PD-1 blockade further enhanced the anti-tumor activity achieved from combination treatment in vitro. The use of checkpoint inhibitors has thus far proven successful in many cancers and their use in ovarian cancer looks promising. The rationale behind using PD-1 blockade in this work was the finding that PD-1 was dramatically upregulated on the surface of T4 cells following co-culture with ovarian tumor cells. This finding was unique to T4 cells and not untransduced cells or other CAR-engineered T cells (P4; targeting prostate specific membrane antigen). The interaction between the ErbB-rich tumor cells and T4 cells determined an increase in PD-1 on the surface of T cells. Additionally, treatment with chemotherapy alone determined an increased surface expression of PD-L1 on SKOV-3 tumor cells.

PD-1 activation by PD-L1 is known to inhibit T cell proliferation and activity. Therefore, we hypothesized that blockade of PD-1 would enhance T cell function and activity in this system. Indeed, this was the case when an anti-PD-1 antibody was used in combination with chemotherapy and T4 immunotherapy. Tumor cell viability in vitro was significantly reduced following PD-1 blockade in immortalized cell lines treated with T4 alone and with both chemotherapy and T4 cells, the latter having a more significant effect. In vivo, similar findings were confirmed with PD-1 blockade in combination with T4 cells and paclitaxel causing a significant regression in tumor burdens. Moreover, PD-1 blockade increased T4 activity as shown by the increase in IFNγ and Granzyme B levels in supernatants tested after treatment in vitro. IFNγ and Granzyme B levels were high when T4 cells were used and were significantly increased following PD-1 blockade. The levels appear to increase with increasing doses of paclitaxel or carboplatin, however, this dose-dependent trend was not significant.

As discussed above, combining traditional therapies with immunotherapies is becoming increasingly popular, especially due to some outstanding successes seen in combinatorial approaches using checkpoint inhibitors. However, the mechanisms which govern synergistic interactions in combination therapies are often unclear.

Both paclitaxel and carboplatin have been shown to induce apoptosis in tumor cells. In fact, DNA damage caused by chemotherapy leads to cleavage and activation of intracellular caspases, initiating a proteolytic cascade and eventually cell death. Reversal of apoptosis can be achieved using the pan-caspase inhibitor Z-VAD, which binds to caspase proteases irreversibly, preventing the initiation of the proteolytic cascade. Treating ovarian tumor cells with chemotherapy and Z-VAD resulted in a reversal of the anti-tumor activity observed following treatment with chemotherapy. When Z-VAD was used with chemotherapy and T4 cells, there was a partial, yet significant reversal in the reduction seen in tumor cell viability. The reversal was not complete, i.e., back to baseline, suggesting that caspase induction, or indeed apoptosis, was not the sole mechanism but was definitely contributing to the synergistic effect of our combination therapy.

Another potential mechanism we investigated was the role of autophagy and particularly M6PR, in our system. We showed that M6PR is upregulated on the tumor cell surface following chemotherapy treatment in vitro, both by flow cytometry and immunofluorescence. Functionally, co-culturing T4 cells with tumor cells following chemotherapy resulted in an increase in intracellular tumor Granzyme B, possibly implicating M6PR as a ‘gateway’ for Granzyme B to enter the tumor.

It has been suggested that following chemotherapy M6PR is shuttled to the tumor cell surface rather than being synthesized de novo [27]. To investigate if this was the case in our system, we looked at its mRNA and protein levels in SKOV-3-luc cells following chemotherapy treatment. Tumor mRNA levels of M6PR did not change which, together with our immunofluorescence and flow cytometry findings, contribute to the hypothesis of a chemotherapy-induced shuttling of M6PR rather than increased *de novo* synthesis. To determine whether M6PR shuttling is involved in the synergistic interaction between chemotherapy and T4 immunotherapy, we indirectly blocked its surface upregulation using 3-MA—an autophagy inhibitor which blocks the formation of autophagosomes and subsequent release of M6PR to the tumor cell surface. Treating SKOV-3-luc cells with 3-MA resulted in a decrease in the surface M6PR expression, but not total levels. When used in combination with chemotherapy and T4 cells, 3-MA indeed resulted in a significant reversal in the anti-tumor effect seen with combination therapy. Combination chemotherapy and 3-MA in the absence of T4 cells did not result in a change in tumor cell viability. Furthermore, intracellular Granzyme B levels were significantly reduced in tumor cells treated with chemotherapy and 3-MA followed by T4 cells, suggesting that the “gateway” had been closed. These findings strongly implicate M6PR shuttling as another key process which significantly contributes to the synergistic effect in our chemo-immunotherapy approach.

Finally, it is known that paclitaxel exerts its anti-neoplastic effect by disruption of the tumor cell cycle at the G2/M phase. Carboplatin, as well as causing DNA adducts and inter-and intra-strand links, also causes G2/M disruption. Therefore, we hypothesized that such cell cycle disruption at G2/M phase might be involved in sensitizing tumor cell to T4 immunotherapy.

To test this hypothesis, we induced G2/M arrest in tumor cells using Thiostrepton, and assessed if its combination with T4 immunotherapy had any enhanced anti-tumor effect. Thiostrepton is a cyclic peptide antibiotic which inhibits protein synthesis by blocking the binding of GTP to the 50S ribosomal subunit, and a specific inhibitor of the G2/M regulator FOXM1. Similar to chemotherapy, treatment with Thiostrepton resulted in a dose-dependent decrease in tumor cell viability. When combined with T4 cells, a significantly enhanced anti-tumor effect was observed. It is therefore likely that G2/M arrest contributes to sensitizing the tumor cells to immunotherapy. However, the reason as to why this may be happening remains unclear and requires further investigation.

In conclusion, our work demonstrates a therapeutic advantage to using combination chemotherapy and adoptive T cell therapy in the treatment of ovarian cancer. Additionally, it provides a rationale for using checkpoint inhibitors, in particular PD-1 blockade which can further enhance the anti-tumor therapeutic effect of combination therapy. This most likely occurs by counteracting the increased upregulation of PD-L1/PD-1 determined by chemotherapy and T cell–tumor contact, respectively, which we showed in our system. Moreover, we show how our three-agent approach, combining traditional chemotherapy, T4 immunotherapy and PD-1 blockade, is successful in vivo, in our ovarian cancer murine model.

It is evident that low-dose chemotherapy has a sensitizing effect on tumor cells, allowing better killing by cytotoxic lymphocytes. We have identified three mechanisms all of which significantly contribute to the synergistic interaction of chemo-immunotherapy against ovarian cancer cells. We conclude that the mechanism of such synergy is multifaceted and is likely to involve other pathways which still remain to be investigated. Targeting these pathways may prove beneficial to maximize the benefit from chemo-immunotherapy.

Though our findings are based on established ovarian cancer cell lines used in in vitro and in vivo models, they give insights into the mechanisms of synergy between immunotherapy and chemotherapy; these would be expected to be similar in play in patients. Further studies in patients who may be on similar combination therapies are required and would add evidence to our findings.

Increasing our understanding of synergistic interactions is essential to better stratify patients and deliver appropriate combination treatments.

## Abbreviations

3-MA: 3-Methyladenine
BLI: Bioluminescence imaging
EOC: Epithelial ovarian cancer
GTP: Guanosine triphosphate
Luc: Luciferase
M6PR: Mannose-6-phosphate receptor
Z-VAD: Carbobenzoxy-valyl-alanyl-aspartyl-[*O*-methyl]- fluoromethylketone

## References

[1] Ferlay J, Shin HR, Bray F, Forman D, Mathers C, Parkin DM (2010). Estimates of worldwide burden of cancer in 2008: GLOBOCAN 2008. Int J Cancer.

[2] Berrino F, De Angelis R, Sant M, Rosso S, Bielska-Lasota M, Coebergh JW, Santaquilani M, group EW (2007). Survival for eight major cancers and all cancers combined for European adults diagnosed in 1995-99: results of the EUROCARE-4 study. Lancet Oncol.

[3] Ozols RF (2000). Management of advanced ovarian cancer consensus summary. Advanced Ovarian Cancer Consensus Faculty. Semin Oncol.

[4] Greenlee RT, Hill-Harmon MB, Murray T, Thun M (2001). Cancer statistics, 2001. CA Cancer J Clin.

[5] Liang XJ, Mukherjee S, Shen DW, Maxfield FR, Gottesman MM (2006). Endocytic recycling compartments altered in cisplatin-resistant cancer cells. Cancer Res.

[6] Godwin AK, Meister A, O’Dwyer PJ, Huang CS, Hamilton TC, Anderson ME (1992). High resistance to cisplatin in human ovarian cancer cell lines is associated with marked increase of glutathione synthesis. Proc Natl Acad Sci USA.

[7] Dabholkar M, Bostick-Bruton F, Weber C, Bohr VA, Egwuagu C, Reed E (1992). ERCC1 and ERCC2 expression in malignant tissues from ovarian cancer patients. J Natl Cancer Inst.

[8] Plumb JA, Strathdee G, Sludden J, Kaye SB, Brown R (2000). Reversal of drug resistance in human tumor xenografts by 2′-deoxy-5-azacytidine-induced demethylation of the hMLH1 gene promoter. Cancer Res.

[9] Rosenberg SA, Restifo NP, Yang JC, Morgan RA, Dudley ME (2008). Adoptive cell transfer: a clinical path to effective cancer immunotherapy. Nat Rev Cancer.

[10] Parente-Pereira AC, Whilding LM, Brewig N, van der Stegen SJ, Davies DM, Wilkie S, van Schalkwyk MC, Ghaem-Maghami S, Maher J (2013). Synergistic chemoimmunotherapy of epithelial ovarian cancer using ErbB-retargeted T cells combined with carboplatin. J Immunol.

[11] John LB, Devaud C, Duong CP, Yong CS, Beavis PA, Haynes NM, Chow MT, Smyth MJ, Kershaw MH, Darcy PK (2013). Anti-PD-1 antibody therapy potently enhances the eradication of established tumors by gene-modified T cells. Clin Cancer Res.

[12] Davies DM, Foster J, Van Der Stegen SJ, Parente-Pereira AC, Chiapero-Stanke L, Delinassios GJ, Burbridge SE, Kao V, Liu Z, Bosshard-Carter L, Van Schalkwyk MC, Box C, Eccles SA, Mather SJ, Wilkie S, Maher J (2012). Flexible targeting of ErbB dimers that drive tumorigenesis using genetically engineered T cells. Mol Med.

[13] Siwak DR, Carey M, Hennessy BT, Nguyen CT, McGahren Murray MJ, Nolden L, Mills GB (2010). Targeting the epidermal growth factor receptor in epithelial ovarian cancer: current knowledge and future challenges. J Oncol.

[14] Anglesio MS, Kommoss S, Tolcher MC, Clarke B, Galletta L, Porter H, Damaraju S, Fereday S, Winterhoff BJ, Kalloger SE, Senz J, Yang W, Steed H, Allo G, Ferguson S, Shaw P, Teoman A, Garcia JJ, Schoolmeester JK, Bakkum-Gamez J, Tinker AV, Bowtell DD, Huntsman DG, Gilks CB, McAlpine JN (2013). Molecular characterization of mucinous ovarian tumours supports a stratified treatment approach with HER2 targeting in 19% of carcinomas. J Pathol.

[15] Ocana A, Vera-Badillo F, Seruga B, Templeton A, Pandiella A, Amir E (2013). HER3 overexpression and survival in solid tumors: a meta-analysis. J Natl Cancer Inst.

[16] Paatero I, Lassus H, Junttila TT, Kaskinen M, Butzow R, Elenius K (2013). CYT-1 isoform of ErbB4 is an independent prognostic factor in serous ovarian cancer and selectively promotes ovarian cancer cell growth in vitro. Gynecol Oncol.

[17] Nars MS, Kaneno R (2013). Immunomodulatory effects of low dose chemotherapy and perspectives of its combination with immunotherapy. Int J Cancer.

[18] Pushkarev VM, Starenki DV, Saenko VA, Pushkarev VV, Kovzun OI, Tronko MD, Popadiuk ID, Yamashita S (2008). Differential effects of low and high doses of Taxol in anaplastic thyroid cancer cells: possible implication of the Pin1 prolyl isomerase. Exp Oncol.

[19] Yeung TK, Germond C, Chen X, Wang Z (1999). The mode of action of taxol: apoptosis at low concentration and necrosis at high concentration. Biochem Biophys Res Commun.

[20] Furness AJ, Vargas FA, Peggs KS, Quezada SA (2014). Impact of tumour microenvironment and Fc receptors on the activity of immunomodulatory antibodies. Trends Immunol.

[21] Hansen JM, Coleman RL, Sood AK (2016). Targeting the tumour microenvironment in ovarian cancer. Eur J Cancer.

[22] Topalian SL, Drake CG, Pardoll DM (2015). Immune checkpoint blockade: a common denominator approach to cancer therapy. Cancer Cell.

[23] Hamanishi J, Mandai M, Konishi I (2016). Immune checkpoint inhibition in ovarian cancer. Int Immunol.

[24] Mitchell MJ, Webster J, Chung A, Guimaraes PP, Khan OF, Langer R (2017). Polymeric mechanical amplifiers of immune cytokine-mediated apoptosis. Nat Commun.

[25] Wu YT, Tan HL, Shui G, Bauvy C, Huang Q, Wenk MR, Ong CN, Codogno P, Shen HM (2010). Dual role of 3-methyladenine in modulation of autophagy via different temporal patterns of inhibition on class I and III phosphoinositide 3-kinase. J Biol Chem.

[26] Zhou J, Li G, Zheng Y, Shen HM, Hu X, Ming QL, Huang C, Li P, Gao N (2015). A novel autophagy/mitophagy inhibitor liensinine sensitizes breast cancer cells to chemotherapy through DNM1L-mediated mitochondrial fission. Autophagy.

[27] Ramakrishnan R, Huang C, Cho HI, Lloyd M, Johnson J, Ren X, Altiok S, Sullivan D, Weber J, Celis E, Gabrilovich DI (2012). Autophagy induced by conventional chemotherapy mediates tumor cell sensitivity to immunotherapy. Cancer Res.

[28] Rodnina MV, Savelsbergh A, Matassova NB, Katunin VI, Semenkov YP, Wintermeyer W (1999). Thiostrepton inhibits the turnover but not the GTPase of elongation factor G on the ribosome. Proc Natl Acad Sci USA.

[29] Kwok JM, Myatt SS, Marson CM, Coombes RC, Constantinidou D, Lam EW (2008). Thiostrepton selectively targets breast cancer cells through inhibition of forkhead box M1 expression. Mol Cancer Ther.

